# Inhibition of resistant triple-negative breast cancer cells with low-dose 6-mercaptopurine and 5-azacitidine

**DOI:** 10.18632/oncotarget.27922

**Published:** 2021-03-30

**Authors:** Balraj Singh, Vanessa N. Sarli, Anthony Lucci

**Affiliations:** ^1^Department of Breast Surgical Oncology, The University of Texas MD Anderson Cancer Center, Houston, Texas, USA; ^2^Morgan Welch Inflammatory Breast Cancer Research Program and Clinic, The University of Texas MD Anderson Cancer Center, Houston, Texas, USA

**Keywords:** resistant TNBC, minimal residual disease, intratumor heterogeneity, breast cancer relapse, metastasis prevention

## Abstract

Highly adaptable breast cancer cells that can opportunistically switch between proliferation and quiescence are often responsible for disease relapse. We have developed a function-based selection strategy for such resistant cells, exemplified by SUM149-MA and FC-IBC02-MA triple-negative breast cancer cells. We have also reported that a lengthy treatment with low-dose 6-mercaptopurine, a clinically useful anti-inflammatory drug, inhibits such resistant cells. To more rigorously test the clinical suitability of 6-mercaptopurine, here we investigated effects of further lowering its dose and the possibility of overcoming resistance to single-drug treatment by combining the drug with another ribonucleoside analog 5-azacitidine. We found that that a lengthy treatment with 1 μM 5-azacitidine, without a significant effect on cell proliferation, sensitized cancer cells to the inhibitory effects of low-dose 6-mercaptopurine. Importantly, treatment for several weeks with low doses of 6-mercaptopurine and/or 5-azacitidine did not render cancer cells resistant to chemotherapeutic drugs doxorubicin or paclitaxel. In fact, the cells became more sensitive to chemotherapeutic drugs upon treatment with 6-mercaptopurine and/or 5-azacitidine. Our analyses of protein markers of epithelial-to-mesenchymal transition indicated that treatments with 6-mercaptopurine and/or 5-azacitidine do not significantly reverse this process in our model. Our results showed that safe drugs such as low-dose 6-mercaptopurine singly or combined with 5-azacitidine, which are suitable for use prior to disease relapse, have a potential of inhibiting highly resistant triple-negative breast cancer cells.

## INTRODUCTION

Breast cancer patients who have minimal residual disease (MRD) after surgery or systemic therapies are at a higher risk of relapse [1–3]. The ability of MRD to efficiently switch between quiescence and proliferation, depending on the challenges in the body, increases likelihood of relapse. With the goal of developing therapies that would halt the progression of MRD to clinical metastases, we have developed a cell culture model of such resistant breast cancer cells. The model involves choosing cancer cell lines established from therapy-resistant breast cancers, such as inflammatory breast cancer (IBC), and subjecting them to prolonged glutamine deficiency to select progenitor-like cancer cells that are highly resistant and can metastasize to multiple organs in nude mice [4–8]. Functional studies and molecular analyses of these adaptable cells have revealed a variety of factors (e.g., genetic mutations, modifications of the epigenome, transcriptome, and proteome) that generate a tremendous cellular heterogeneity and confer survival advantages under various bottlenecks in the body. It is noteworthy that the adaptable cancer cells modeled in our study are resistant to chemotherapeutic drugs; therefore such cells would likely persist after currently used neoadjuvant or adjuvant therapies. Evidence suggests that SUM149-metabolic adaptable (MA) cells are a suitable model of resistant human triple-negative breast cancer (TNBC) cells that can survive bottlenecks in the body, including therapeutic interventions, by opportunistically switching between quiescence and cell proliferation [5, 7, 8].

To explain our experimental strategy for therapeutic evaluation against resistant cancer cells, although our selection protocol for resistant cells is very robust, a majority of the progeny cells would gradually revert back to non-resistant cells in non-selective *in vitro* conditions. Since a majority of relatively sensitive cells are preferentially eliminated first by most therapies, lengthy therapeutic evaluations in cell culture provide more useful information about the resistant subpopulation of cancer cells than rapid cell proliferation assays. Besides a suitable cell culture model of deep intrinsic resistance, other complementary aspect of our drug discovery approach is prioritizing potential therapeutic agents that may be suitable for an early use before clinical metastasis is detected. For this purpose, we rely on the safety-related information resulting from clinical use of such compounds.

For a potential therapy to be suitable at the MRD stage, it must be safe (an important criterion prior to clinical relapse) and disrupt heterogeneous progenitor-like cancer cells that evolve into clinical metastases. Here we evaluated two ribonucleoside analogues, namely 6-mercaptopurine (6-MP) and 5-azacitidine (5-AzaC), at low doses because of their potential to disrupt transcriptome and epigenome in MRD. 6-MP treatment affects cells via mis-incorporation of 6-thioguanine triphosphate into RNA and deoxy-6-thioguanine triphosphate into DNA along with other effects on nucleoside pools and cell signaling. We chose low-dose 6-MP for evaluation in our model of adaptable cancer cells because of 6-MP’s ability to induce and maintain remission in inflammatory bowel disease (IBD) [9] and childhood acute lymphoblastic leukemia [10] (both situations require control of abnormal progenitor cells). Furthermore, we recently reported that a lengthy treatment with 4 μM 6-MP inhibited progenitor-like SUM149-MA cells from proliferating, thus keeping them arrested in quiescence [8]. Although a 4 μM concentration of 6-MP can be considered a low dose in cell culture, because it does not significantly inhibit cell growth during 1 week of treatment, it is not low enough compared with the 6-MP concentration that would be achieved in the body. Therefore, here we investigated the effects of even lower (1 μM) concentration of 6-MP; we evaluated even lower 0.1 μM dose of 6-MP in FC-IBC02 and FC-IBC02-MA cells because of their relatively higher sensitivity to this drug [8]. Additionally, recognizing that 6-MP alone may not adequately inhibit all resistant cancer cells, we also evaluated whether another ribonucleoside analogue, namely 5-AzaC, could enhance the effects of 6-MP. We chose 5-AzaC because it could complement 6-MP’s effects on the transcriptome and epigenome, and—as indicated by many Phase 1 clinical trials—5-AzaC is well tolerated [11]. It has also been shown to stabilize quiescence in cancer cells and to sensitize resistant cancer cells to cell death with an apoptosis-inducing agent in a preclinical model of multiple myeloma [12].

## RESULTS

### Evidence of an altered transcriptome in SUM149-MA cells

**Figure 1:**
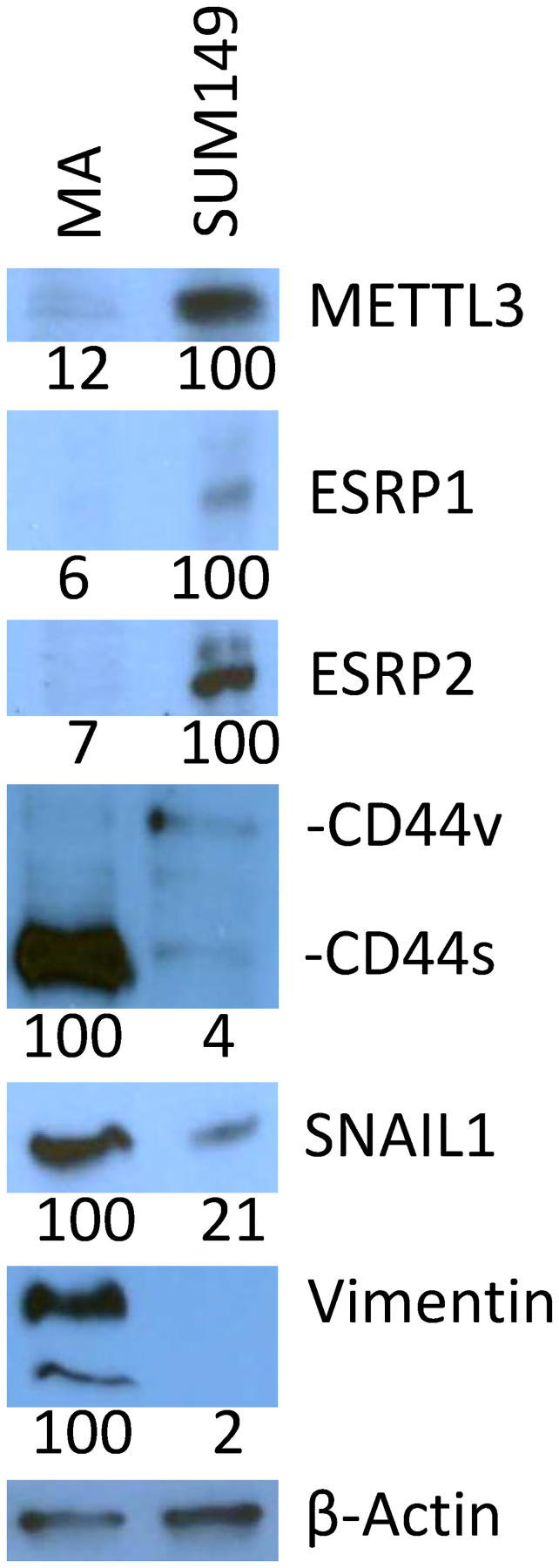
Validation of selected gene expression data with Western blotting. Lower levels of METTL3, ESRP1, ESRP2, and CD44v and higher levels of CD44s, SNAIL1, and vimentin are seen in SUM149-MA cells as compared to parental cell line. Parental SUM149-Luc cells were cultured in glutamine-containing medium with dialyzed FBS (indicated in the figure as SUM149). SUM149-MA cells (MA) were maintained in a glutamine-free medium with dialyzed FBS for 9 passages and then switched to glutamine-containing medium for 5 passages before preparing cell lysates for this analysis. Filters were re-probed with a β-actin antibody to normalize sample loading. The β-actin blot shown here is a re-probe of the CD44 blot. Relative intensities of protein bands, as quantified with the ImageJ software, are shown at the bottom; the values under the CD44 blot are for CD44s.

Our gene expression data suggest that RNA modifications play a major role in adaptability of MA cells [5]. Here, we validated some gene expression changes, which may drive RNA modifications, by Western blotting. N-^6^ methyladenosine (m^6^A) modification in RNA, which influences RNA function in multiple ways, has emerged as a major determinant of cell fate [13, 14]. Alterations in m6A regulation of RNA may be involved in breast cancer progression [15]. We found that MA cells had dramatically lower levels of METTL3 m^6^A methyltransferase as compared with parental SUM149 cells (Figure 1). This result, along with our previous result showing an increase in m^6^A demethylase FTO [6], would support a transcriptome with low m^6^A. The m^6^A modification is recognized by the reader proteins, and these interactions may influence other modifications in RNA—both base modifications and alternative RNA splicing [16, 17].

Epithelial to mesenchymal transition (EMT) is an important manifestation of cell plasticity. MA cells have indicators of high EMT such as high ZEB1, low GRHL2, low ESRP1, and high CD44s [5, 8]. Here we validated a finding from gene expression data that MA cells have a high level of another EMT indicator, namely SNAIL1 (Figure 1). The mesenchymal marker vimentin, which is absent in parental cells, is dramatically induced in MA cells [8]. Here we also show that MA cells have a low level of ESRP2 in addition to low ESRP1, together indicating that these changes may drive alternative RNA splicing, thus changing the cell fate from epithelial to mesenchymal. One of the direct results of this alternative splicing is a shift in CD44 isoforms from CD44v to CD44s (Figure 1).

### Sensitization of SUM149 and SUM149-MA cells to 6-MP with 5-AzaC treatment

When we treated SUM149 and SUM149-MA cells with 1 μM 6-MP, there was no significant growth inhibition at 7 days (Supplementary Figure 1). However, as we passaged cells and continued treatment for several weeks, we observed significant growth inhibition, which was more pronounced in SUM149-MA cells than in parental SUM149 cells. These results are similar to the results obtained with 4 μM 6-MP [8], except the time required for growth inhibition is longer as the dose is lowered. A lower level of inhibition is expected with a lower dose. What was encouraging is that we did not observe the emergence of resistant fast-growing colonies as one does in cases of treatment with chemotherapeutic drugs [4].

**Figure 2:**
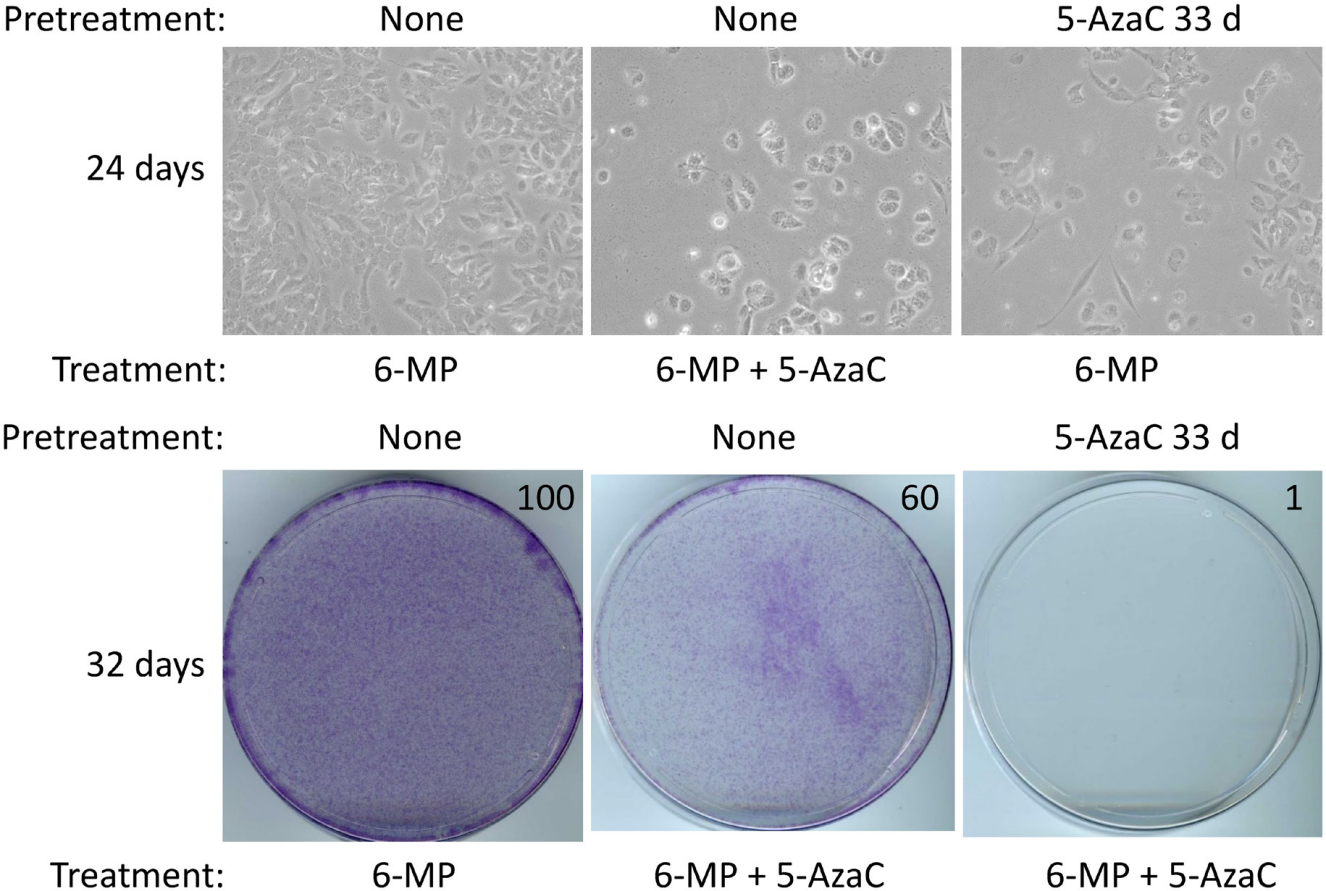
5-AzaC treatment sensitizes SUM149 cells to low-dose 6-MP. Both 5-AzaC and 6-MP were used at 1 μM dose. Top left: treatment with 6-MP alone caused very little growth inhibition. Top middle: co-treatment with 5-AzaC and 6-MP caused significant growth inhibition in SUM149-Luc cells. Top right: cells were first pretreated with 5-AzaC for 33 days (5-AzaC is non-cytotoxic at this dose), allowed to recover in drug-free medium for 7 days, and then treated with 6-MP. For all three panels, cells (following pretreatment, if any) were treated with the indicated drugs in parallel for total 24 days, involving a passage at day 8. Co-treatment or pretreatment with 5-AzaC significantly increased the 6-MP mediated growth inhibition. Representative images taken at 10× magnification are shown. Bottom: The cells were treated with the indicated drugs in parallel for a total 32 days (last cell passage at day 24). Then they were stained with crystal violet. Relative cell mass present on the dishes, as measured by solubilizing the dye and reading absorbance at 570 nm, is shown on the top right of photographs of dishes.

5-AzaC has emerged as a promising agent with a therapeutic potential at MRD stage [12, 18, 19]. In a preliminary screen of compounds that would inhibit MA cells, a 27 days treatment with 1 μM 5-AzaC sensitized MA cells to doxorubicin [5]. Here we evaluated whether 5-AzaC would affect resistant cells in our model, thus sensitizing them for 6-MP’s action. We used a low, non-cytotoxic dose of 5-AzaC (1 μM) that does not significantly inhibit cell growth even after several weeks of treatment (see Supplementary Figure 1 for photographs of stained dishes after 7 days treatment). We first evaluated 5-AzaC in SUM149 cells, which are more resistant to 6-MP than are MA cells [8]. We found that after 24–32 days of co-treatment with 5-AzaC and 6-MP, there was significant growth inhibition compared with cells treated with 6-MP alone (Figure 2). We also evaluated whether 33 days of pretreatment with 5-AzaC sensitized cells to 6-MP’s action. In this manner, we found that 5-AzaC pretreated cells were significantly inhibited with 6-MP in 24–32 days as compared with control cells treated with 6-MP in parallel (Figure 2).

**Figure 3:**
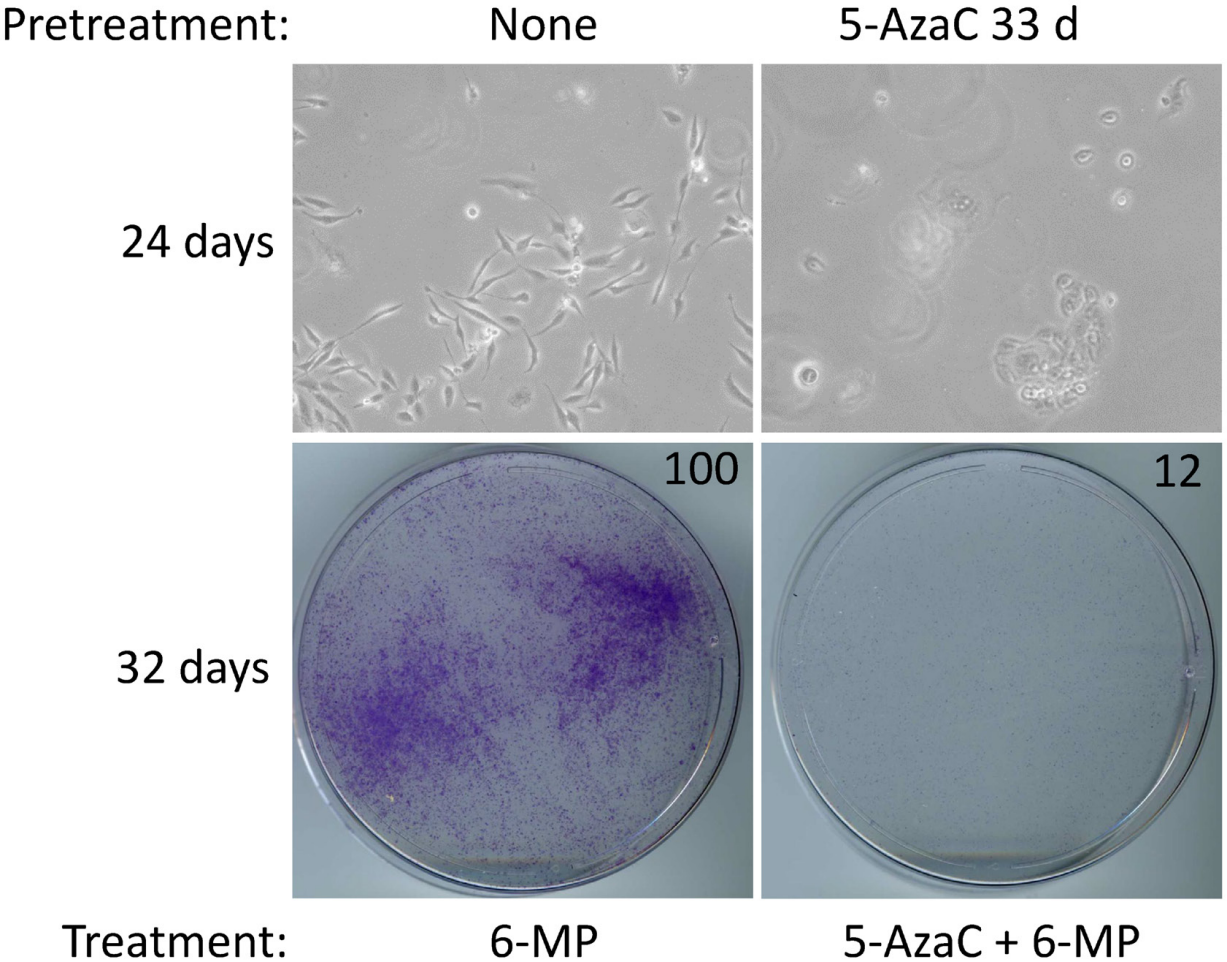
5-AzaC pretreatment sensitizes SUM149-MA cells to low-dose 6-MP. Both 5-AzaC and 6-MP were used at 1 μM dose. Top left: treatment with 6-MP alone caused growth inhibition in SUM149-MA cells. Top right: cells were first treated with 5-AzaC for 33 days (5-AzaC is non-cytotoxic at this dose), allowed to recover in drug-free medium for 7 days, and then treated with 5-AzaC plus 6-MP. For both panels, cells (following pretreatment, if any) were treated with indicated drugs in parallel for total 24 days, involving a passage at day 8. Pretreatment with 5-AzaC significantly increased the growth inhibition mediated by co-treatment with 6-MP and 5-AzaC. Representative images taken at 10× magnification are shown. Bottom: The cells were treated with the indicated drugs in parallel for a total 32 days (last cell passage at day 24). Then they were stained with crystal violet. Relative cell mass present on the dishes, as measured by solubilizing the dye and reading absorbance at 570 nm, is shown on the top right of photographs of dishes.

Although cell proliferation in MA cells is inhibited with 4 μM 6-MP, a small subpopulation of cells persists in quiescence, which can begin proliferating after 6-MP is withdrawn [8]. Therefore, we investigated whether 5-AzaC treatment impacts 6-MP–resistant SUM19-MA cells. We compared SUM149-MA cells treated with 6-MP alone for 24–32 days (control) with cells co-treated with 5-AzaC and 6-MP. We found that a co-treatment did not significantly inhibit SUM149-MA cells beyond the inhibition achieved with 6-MP alone (data not shown). However, when the cells were pretreated with 5-AzaC for 33 days before co-treating them with 5-AzaC and 6-MP for 24–32 days, we observed dramatic growth inhibition and morphological evidence of severe cytotoxicity in these cells (Figure 3). This result, along with the result obtained with SUM149 cells (Figure 2), indicates the potential of 5-AzaC in overcoming two different types of resistance (represented in parental and MA cells) to 6-MP monotherapy.

Next, we determined whether the lengthy treatments with 6-MP and/or 5-AzaC, which inhibit treatment-resistant cancer cells, lead to changes in EMT phenotype. We determined relative levels of several proteins, which are indicators of EMT in our system, by Western blotting. To conduct these studies, we treated SUM149-MA cells with 5-AzaC several different ways (different lengths of treatment, different times of recovery without drug after treatment, and a treatment-recovery-treatment regimen) before Western blotting. A Western blot for ESRP1, ESRP2, CD44s, SNAIL1, and vimentin is shown (Supplementary Figure 2). We found that none of these treatment regimens had any significant effect on EMT markers. Treatment of SUM149-MA cells with 5-AzaC for 28 days followed by a 6-MP treatment for 24 days also failed to affect the EMT indicators (Supplementary Figure 2). These results indicate that a sensitization of resistant cancer cells with 5-AzaC treatment does not involve a reversal of high EMT in our model.

### Cancer cells remaining after 6-MP and 5-AzaC treatments are sensitive to cytotoxic drugs

**Figure 4:**
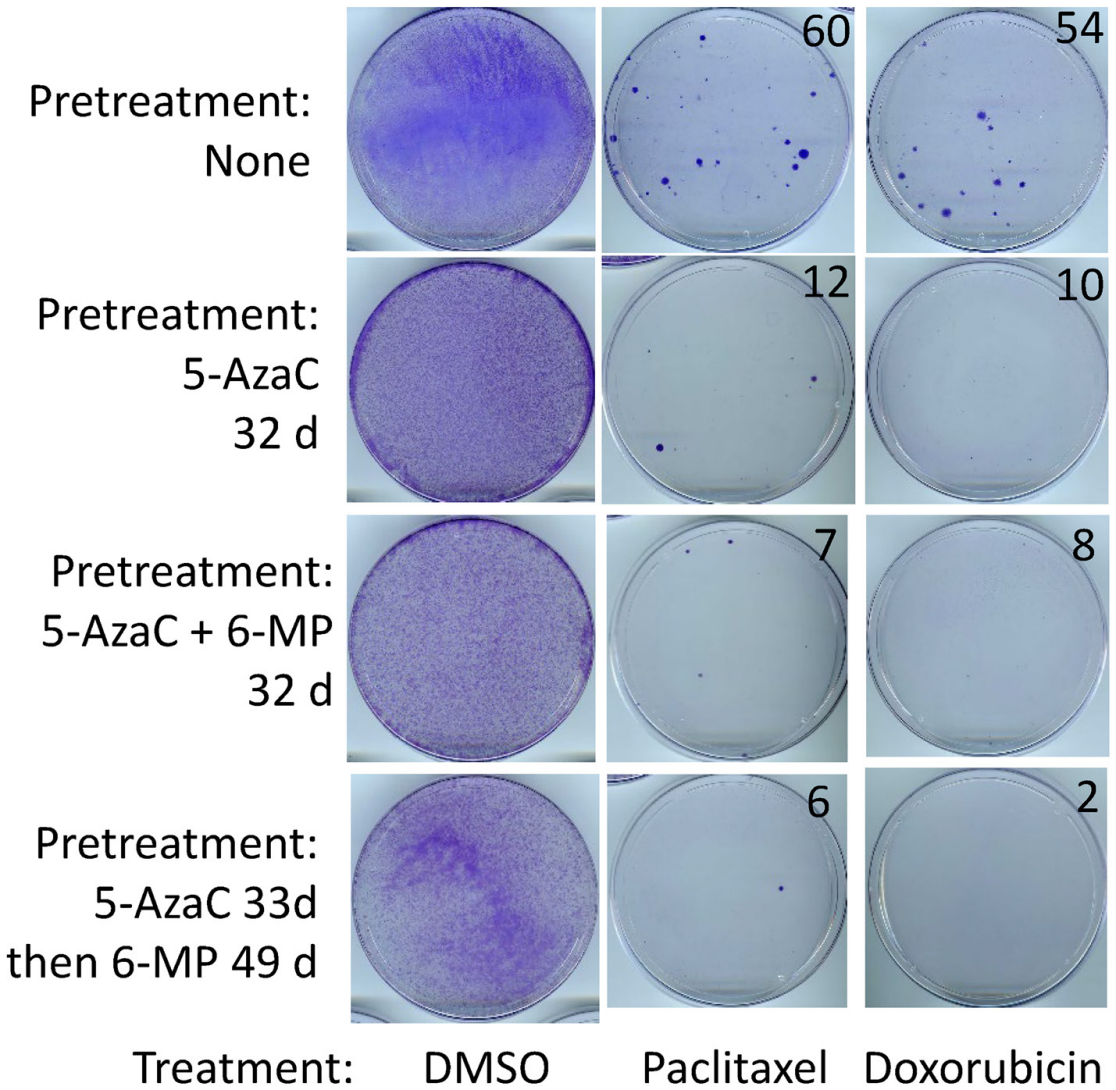
SUM149 cells surviving 5-AzaC/6-MP treatments are sensitive to cytotoxic drugs. After lengthy treatments with the indicated drugs (both 5-AzaC and 6-MP at 1 μM) shown on left, surviving SUM149-Luc cells were allowed to recover. They were then treated in parallel with 5 nM paclitaxel or 50 nM doxorubicin for 6 days, and then resistant cells were allowed to recover and grow into colonies for 17 days before staining with crystal violet. Cells treated with DMSO solvent in parallel served as controls. DMSO-treated control plates were stained after 6 days. Recovery period for dishes shown in the second and third panels from top before treatment with chemotherapeutic drugs: 15 days each; for dishes shown in bottom panel: 7 days between 5-AzaC and 6-MP treatments. The number of colonies, counted manually on full-size images, are shown on the top right of photographs of dishes. Representative cell cultures are shown.

A clinically relevant question in the context of a heterogeneous disease is whether the cancer cells remaining after 6-MP and 5-AzaC treatments have become more resistant than untreated cells. To address this issue, the treated cells were allowed to recover in a drug-free medium and then subjected to treatment with paclitaxel or doxorubicin in parallel with untreated control cells. These assays were carried out in standard manner, which involves first elimination of relatively sensitive cancer cells followed by a growth of remaining resistant cells into colonies. We have shown previously that the parental cells remaining after a treatment with 6-MP alone are not more resistant than untreated cells [8]. To account for the possibilities of different mechanisms of potential resistance under different treatments, we analyzed parental SUM149 cells that had been exposed for several weeks to 5-AzaC alone, 5-AzaC and 6-MP together, or 5-AzaC followed by 6-MP in a sequence. We found that the cancer cells remaining after all these treatments were less resistant to chemotherapeutic drugs than untreated cells, as indicated by the number of colonies (Figure 4).

**Figure 5:**
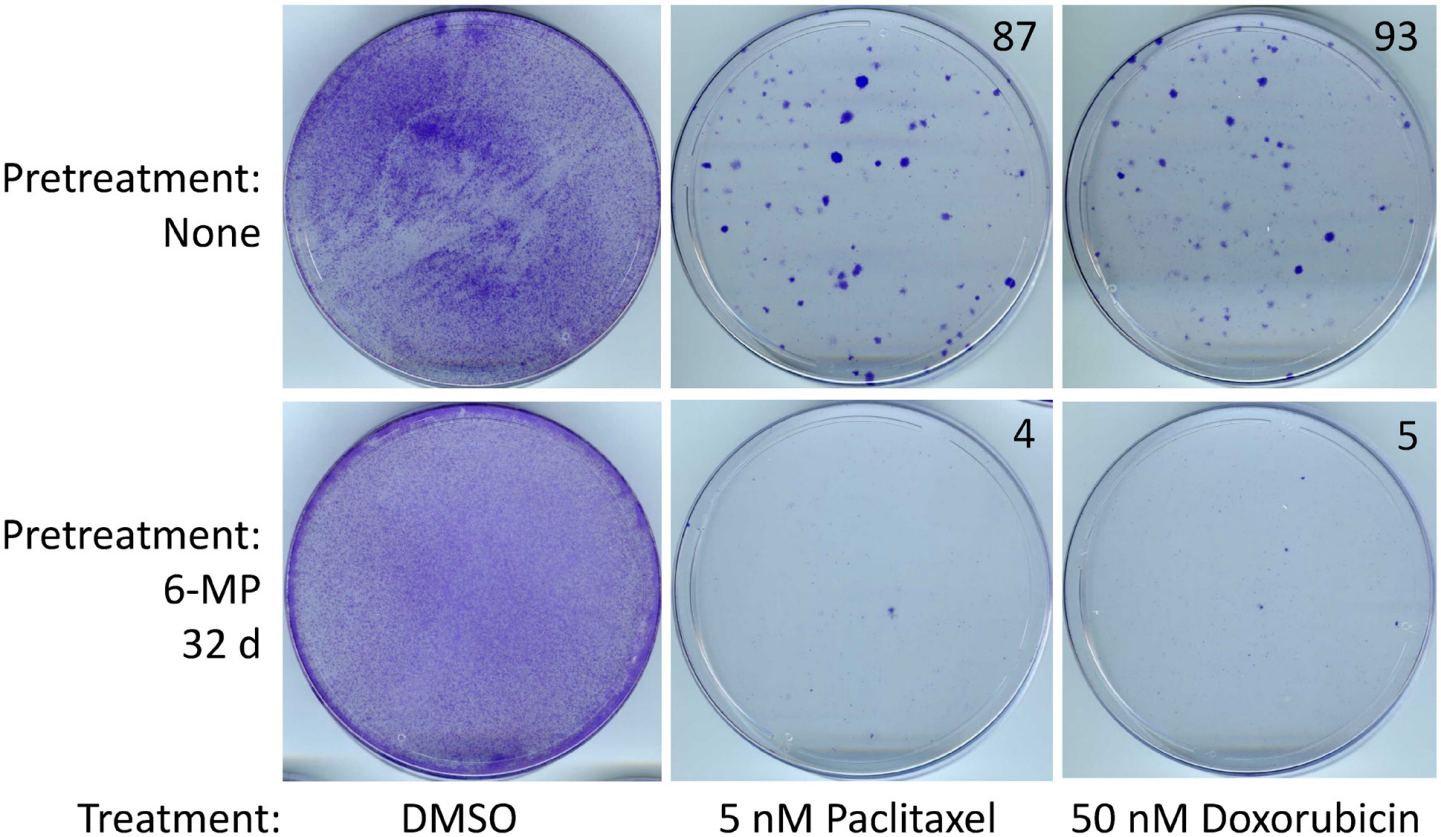
SUM149-MA cells surviving a 6-MP treatment are sensitive to chemotherapeutic drugs. After a 32 days treatment with 2 μM 6-MP, surviving cells were allowed to recover in a drug-free medium for 33 days. Following this, cells were treated in parallel with 5 nM paclitaxel or 50 nM doxorubicin for 6 days and then allowed to recover and grow into colonies for 17 days before staining with crystal violet. Cells treated with DMSO solvent in parallel served as controls. DMSO treated control plates were stained after 6 days. The number of colonies, counted manually on full-size images, are shown on the top right of photographs of dishes. Representative cell cultures are shown.

Next, we examined the chemotherapeutic resistance in the rare SUM149-MA cells that survive 32 days of treatment with 2 μM 6-MP. We found that these cells are less resistant to paclitaxel and doxorubicin than untreated SUM149-MA cells (Figure 5). Because SUM149-MA cells are more sensitive to 6-MP than the parental SUM149 cells, we did not analyze the effects of combination treatments with 5-AzaC on sensitivity to chemotherapeutic drugs in these cells. Together, these results suggest that exposure to low-dose 6-MP and/or 5-AZaC does not increase intrinsic resistance in cancer cells.

### Inhibition of FC-IBC02-MA cells with a co-treatment with low-dose 6-MP and 5-AzaC

**Figure 6:**
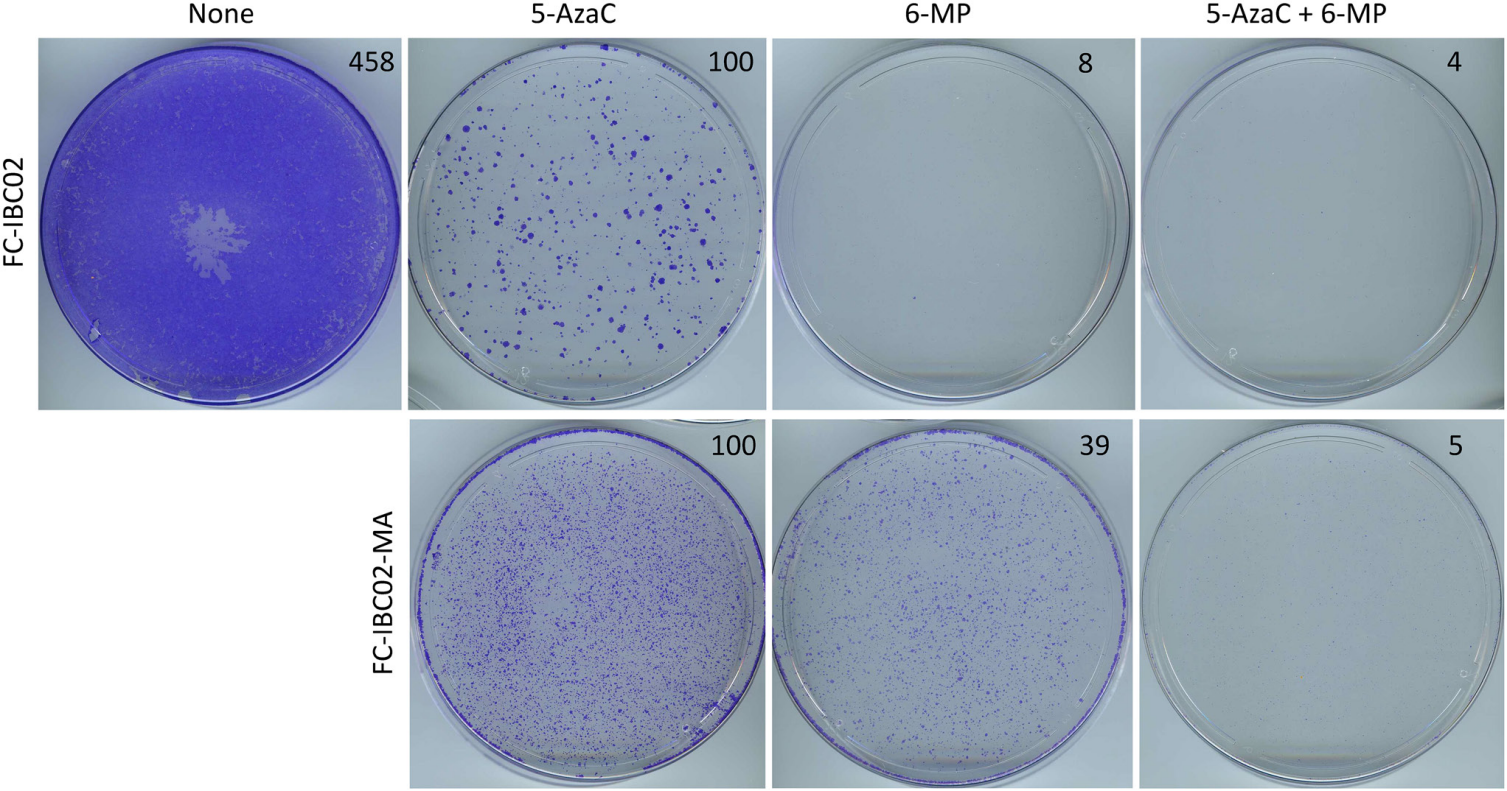
Inhibition of FC-IBC02 and FC-IBC02-MA cells with low-dose 6-MP and 5-AzaC. Top: FC-IBC02 cells were treated with 1 μM 5-AzaC, 0.1 μM 6-MP, or both drugs at these concentrations as indicated for 14 days, following which the remaining cells were allowed to recover in the medium without any drugs for 13 days before staining with crystal violet. A control untreated dish where cells grew to confluency is also shown. Bottom: FC-IBC02-MA cells were treated with 1 μM 5-AzaC, 0.1 μM 6-MP, or both drugs at these concentrations as indicated for 7 days, following which the remaining cells were allowed to recover in the medium without any drugs for 6 days before staining with crystal violet. Relative cell mass present on the dishes, as measured by solubilizing the dye and reading absorbance at 570 nm, is shown on the top right of photographs of dishes.

To address whether the resistant cancer cells derived from other cell lines may also be inhibited with a lengthy treatment with low-dose 6-MP and 5-AzaC, we used a pair of resistant cell lines, namely FC-IBC02 and FC-IBC02-MA. FC-IBC02 has been established from a therapy-resistant triple-negative IBC in a more recent era [20]. We have derived its metabolically adaptable version FC-IBC02-MA that is more resistant to chemotherapeutic drugs than its parental cell line, by selection in a glutamine-free medium analogous to the selection of SUM149-MA cells [7, 8]. Treatment with 1 μM 5-AzaC for 14 days resulted in a modest inhibition of FC-IBC02 cells as revealed by recovery of the remaining cells in a drug-free medium for 13 days followed by their crystal violet staining, which showed a large number of colonies (Figure 6). In contrast, a parallel treatment with a low dose of 0.1 μM 6-MP for 14 days followed by recovery and staining showed a near complete cell inhibition (Figure 6). Not unexpectedly, a combination of both drugs also resulted in a near complete growth inhibition of FC-IBC02 cells (Figure 6).

We obtained a more interesting result upon evaluation of these drugs in highly resistant FC-IBC02-MA cell line. While the treatment with either 1 μM 5-AzaC or 0.1 μM 6-MP for 7 days followed by a 6 days recovery of remaining cells in a drug-free medium and then staining showed a relatively weak growth inhibition (as indicated by a large number of colonies in both dishes), a parallel treatment with a combination of both drugs showed a near complete growth inhibition (compare dishes shown in Figure 6). These results suggest that 5-AzaC and 6-MP may cooperate in inhibiting highly resistant subpopulations of cancer cells exemplified by the cell lines investigated in this study.

## DISCUSSION

Our drug discovery approach focusing on a difficult phenotype in cancer (opportunistic switching between quiescence and proliferation in cancer cells) is specifically designed for high-risk disease. Our logic is that if a safe therapy would work with a high-risk group, it would be easier to expand to patients who are not at high risk of relapse. On the other hand, if a therapy barely works in a low-risk, relatively homogeneous disease, it would have a little chance in high-risk heterogenous disease. In addition, since our approach focuses on a phenotype that governs resistance in a variety of common and rare cancers, its success can be easily extended to other resistant cancers.

### A cell culture model of resistant breast cancer cells

Arguably, cancer evolution in the human body is restrained by various physiological forces while stem-like, slow-cycling cancer cells continue to persist. While it is not possible to optimally model such cells in xenograft mouse models, a cell culture system offers a flexibility in modeling this important phenotype, i.e., cancer cells that have a high potential to opportunistically switch between quiescence and proliferation. Since it is not feasible to model body-like conditions in preclinical models, our strategy to mitigate this limitation is to rely on therapeutic agents that have undergone extensive testing in humans, thus proving their safety and any additional beneficial attributes such as a potential to favorably modulate chronic inflammation and immunity. Any drugs that have shown a potential of remission in any cancer (common or rare) through testing in patients, would have a priority in our approach.

SUM149-MA cells are noteworthy because they are so resistant/adaptable, endowed with a variety of mechanisms for generating a high diversity, that no therapy can eradicate them all in our model. These embryo-like cells appear to have a variety of functionally interconnected, adaptable, multicomponent modules that allow them to opportunistically switch between quiescence and cell proliferation under all challenges. It is reasonable that, although specific molecular alterations behind cancer cell adaptability may differ from cell to cell and from tumor to tumor, broad commonalities exist. For example, a reliance on altered transcriptome may be a common feature of cancer cell adaptability.

### Basis of RNA-targeting strategy in resistant cancer cells

Besides the results presented here in support of altered transcriptome in resistant MA cells, these cells have several other alterations in expression of genes involved in RNA modifications and RNA function including RNA editing enzymes ADARB1, ADARB2 (that perform A to I editing), 3-methyl cytidine methyltransferase METTL6, pseudouridylate synthase, RNA splicing factors (SLU7, SF3B3, RBPMS, RBPMS2), tRNA base modifications (phosphorylation, γw), tRNA splicing endonucleases TSEN2 and TSEN54, small-subunit processome component, RNA-binding proteins affecting their fate (RBM47, QKI), and ribosomal proteins [5]. The MA cells also contain a variety of alterations in miRNAs and long noncoding RNAs that are highly reliant on structural features of RNA [5]. Taken together, these results suggest that a multitude of mechanisms may alter the transcriptome in MA cells, and strategies to target MA cells’ transcriptome may inhibit them.

Since alterations in transcriptome via RNA modifications (e.g., base modifications and alternative splicing) is a dominant feature of adaptable cancer cells, we have chosen to interfere with this feature. A common approach would be to target specific drivers, e.g., some components of the machineries that alter the transcriptome. This approach is unlikely to work in a heterogeneous disease such as TNBC. Specifically in the transcriptome, many compensatory mechanisms may allow development of resistance to inhibition of a single driver, e.g., the effects of an FTO demethylase inhibitor may be countered by adjusting the level and/or activity of METTl3 methylase or other components in multiprotein complexes regulating RNA methylation/demethylation in cancer cells [13]. Because targeting specific molecular drivers, whose actions are context-dependent, may not be a good strategy for halting progression of heterogeneous multiclonal cancers, now we are focusing more on agents such as 6-MP that may interfere with several strategies that adaptable cancer cells may employ.

In this study we chose to interfere with abnormal transcriptome in resistant cancer cells with ribonucleoside analogs, although there are other possible ways for targeting transcriptome. A good example would be spliceosome-targeted small molecule inhibitors that interfere with RNA splicing, leading to accumulation of double-stranded RNA, which triggers antiviral immune response in TNBC cells [21]. What remains to be determined is which transcriptome-targeted therapies would provide maximum efficacy against resistant cancer cells, while sparing normal cells in the body.

Overall, our results provide a proof of concept that 6-MP may be suitable as a novel adjuvant therapy for TNBC that has a high risk of relapse, due to poor prognosis MRD that has the capability of advancing to clinical metastasis. It may be effective at a safe low dose, and it may not increase the risk of unwanted resistance to other therapies. We hope that a low activity of 6-MP and methotrexate observed in a recent phase II multi-institutional clinical trial in UK, involving BRCA-defective advanced breast cancer or platinum-resistant ovarian cancer patients, who had progressed after ≥ 1 previous line of chemotherapy [22], will not discourage clinical trials to evaluate low-dose 6-MP at a more treatable MRD stage in the future.

### Low-dose 6-MP as a potential therapy for halting relapse in high-risk TNBC

Our studies suggest that low-dose 6-MP, which is a purine analogue and very effective in maintaining remission in IBD [9], inhibits highly adaptable TNBC cells in our model, presumably by disrupting their transcriptome and epigenome. In addition to the direct effects on resistant cancer cells, 6-MP could potentially modulate the immune system toward a healthy state (analogous to its action in IBD), to control residual disease. In fact, this is the main reason we chose to evaluate 6-MP with an intent for repurposing a safe drug that may be capable of combating highly resistant cancer cells while simultaneously inhibiting the low-level chronic inflammation that is common in advancing cancers. Although we cannot assess immune effects in cell culture, we can rely on decades of experience in treating IBD with 6-MP for this knowledge. Briefly, cancer and chronic inflammation support each other, thus creating a vicious cycle. Therefore, a therapeutic agent such as 6-MP that may target both cancer cells and inflammation would be optimal.

We suggest that low dose 6-MP and other drugs that would complement 6-MP’s action, such as 5-AzaC, could be suitable for preventing recurrence and metastasis in high-risk breast cancers. 6-MP could be taken lifelong if it is necessary for maintaining a long-term remission. Other potential candidates for combination therapy with 6-MP would be HDAC inhibitors, which are being tested for their efficacy to overcome therapy resistance in various cancers. In this regard, we have reported that a 1-week treatment with sodium valproate or sodium butyrate, which are inhibitors of class I and class IIa HDACs, respectively, sensitizes SUM149-MA cells to chemotherapeutic drugs [5].

Finally, because immune checkpoint blockade therapy is likely to become common in both neoadjuvant and adjuvant settings in a heterogeneous cancer such as TNBC, we must consider any new therapy in this new context. A common hurdle with the immune checkpoint therapy is severe autoimmune reaction toxicities [23]. In some cases, such severe toxicities can be managed with anti-inflammatory agents like TNF antibodies. When such therapies are given in the setting of metastasis, they may be combined with other therapies such as cytotoxic agents, which may also adversely affect immunity. The ideal solution to these complex issues would be to intervene with immune checkpoint blockade sooner (before clinical metastasis) and further enhance the chance of its success by proactively managing severe autoimmune toxicities. Based on a large body of knowledge resulting from 6-MP use in treating IBD [9], low-dose 6-MP may be an ideal candidate for this purpose. Although therapies like TNF antibodies are useful in managing the acute phase of IBD, 6-MP has been a mainstay for several decades for keeping the disease in remission. This speaks to the value of 6-MP in suppressing chronic inflammation for a long period. Importantly, unlike other drugs that relieve symptoms quickly, 6-MP takes several weeks to demonstrate its effects in IBD. Furthermore, 6-MP and its prodrug azathioprine are also prescribed off-label to suppress chronic inflammation in several other autoimmune diseases, e.g., rheumatoid arthritis, lupus nefritis, autoimmune hepatitis, neuromyelitis optica, myasthenia gravis and multiple sclerosis. Applying the lessons from IBD treatment to the setting cancer treatment, low-dose 6-MP followed by immune checkpoint blockade could be a good way of limiting severe autoimmune toxicities, thereby increasing the chances of success with these promising therapies.

## MATERIALS AND METHODS

### Cell lines and drugs

We have previously described the cell lines and their culture conditions. The cell lines included a well-characterized triple-negative IBC cell line SUM149, its firefly luciferase-transfected version SUM149-Luc [24], and a metabolically adaptable version SUM149-MA [4–8]. The SUM149-MA cell line was derived from rare cells (0.01% cells in population) in SUM149-Luc cell line that survived in quiescence for 3–4 weeks in the absence of exogenous glutamine, and then began proliferating indefinitely. The main reason for using luciferase-transfected cell lines is that a lot of background data obtained with these cell lines, including xenograft studies in nude mice and gene expression data [4–8], forms the basis of this study. The FC-IBC02 cell line, originally developed by Massimo Cristofanilli [20], was cultured in Dulbecco modified Eagle medium (DMEM) supplemented with 10% FBS. Selection of the rare MA cell variants from FC-IBC02 cell line was similar to the selection of the SUM149-MA variants, in glutamine-free medium supplemented with 10% dialyzed FBS [4, 7].

6-MP, 5-AzaC, paclitaxel, and doxorubicin were purchased from Sigma-Aldrich (St. Louis, MO, USA). 6-MP was dissolved in 0.1 M NaOH, 5-AzaC was dissolved in Dulbecco’s phosphate buffered saline, and paclitaxel and doxorubicin were dissolved in dimethyl sulfoxide (DMSO). Equal volumes of the solvents without drugs were added to the control dishes. Solvent volume was 0.04% of the volume of the culture medium.

### Western blotting

We performed Western blotting, involving detection of protein bands as enhanced chemo-luminescence signal on X-ray films, as described previously [25]. The following primary antibodies were used for protein detection: anti-METTL3 (catalog number PA5-28178 ThermoFisher Scientific, Waltham, MA, USA), anti-ESRP1 (catalog number GTX131373, GeneTex, Irvine, CA, USA), (catalog number ab155227, Abcam, Cambridge, MA, USA), anti-CD44 (catalog number MAB7045, R&D Systems, Minneapolis, MN, USA), anti-SNAIL1 (catalog number 3895, Cell Signaling Technology, Danvers, MA, USA), and anti-vimentin (catalog number 3932, Cell Signaling Technology). Each Western blot was performed at least twice; representative blots are shown. We quantified relative intensities of protein bands detected on X-ray films with ImageJ software (National Institutes of Health, Bethesda, MD, USA).

### 5-AzaC treatment mediated sensitization of SUM149 and MA cells to 6-MP

We evaluated low-dose 6-MP through lengthy (several weeks) treatments in cell culture as previously described [8]. To assay 5-AzaC’s capacity in overcoming resistance to 6-MP, we treated cells with both the drugs simultaneously for several weeks. Alternatively, we first pretreated cells with 5-AzaC for 33 days before treating them with 6-MP alone or with both drugs. We documented the effects of these treatments on cell growth and cell morphology. At the end of drug treatments, we stained the dishes with crystal violet, and photographed them. For a quantitation of relative cell mass on the stained dishes, we counted the colonies. In instances where it was not feasible to count colonies since cells were not growing as isolated colonies, we estimated relative cell mass from the concentration of crystal violet dye. To perform this, we dissolved the dye by incubating in 10% acetic acid for 5 hours at room temperature on a bench rocker and measured its optical density at 570 nm [26].

### Assay of relative resistance to paclitaxel and doxorubicin

To determine whether a lengthy treatment with 5-AzaC or 6-MP affected the sensitivity of cells to chemotherapeutic drugs, we first allowed drug-treated cells to recover for a few days and then passaged them. We treated these drug-treated cells in parallel with the control vehicle-treated cells for 6–7 days with predetermined concentrations of chemotherapeutic drugs (5 nM paclitaxel or 50 nM doxorubicin) expected to kill 99% of proliferating cells. We then removed the chemotherapeutic drugs and allowed surviving cells to form colonies for 2 to 4 weeks. Colonies were stained with crystal violet and counted for quantitation of data.

## SUPPLEMENTARY MATERIALS



## Abbreviations

5-AzaC: 5-azacitidine
6-MP: 6-mercaptopurine
DMSO: dimethyl sulfoxide
EMT: epithelial to mesenchymal transition
ESRP1: epithelial splicing regulatory protein 1
FBS: fetal bovine serum
HDAC: histone deacetylase
IBC: inflammatory breast cancer
IBD: inflammatory bowel disease
MA: metabolically adaptable
MRD: minimal residual disease
TNBC: triple-negative breast cancer
