# Supplementary material for: Inhibition of resistant triple-negative breast cancer cells with low-dose 6-mercaptopurine and 5-azacitidine
Source: Oncotarget. 2021 Mar 30;12(7):626–37. doi: 10.18632/oncotarget.27922 (PMC8021029; doi:10.18632/oncotarget.27922)
Supplement: Supplementary file 1 [file oncotarget-12-626-s001.pdf]

Inhibition of resistant triple-negative breast cancer cells with low-dose 6-mercaptopurine and 5-azacitidine

SUPPLEMENTARY MATERIALS

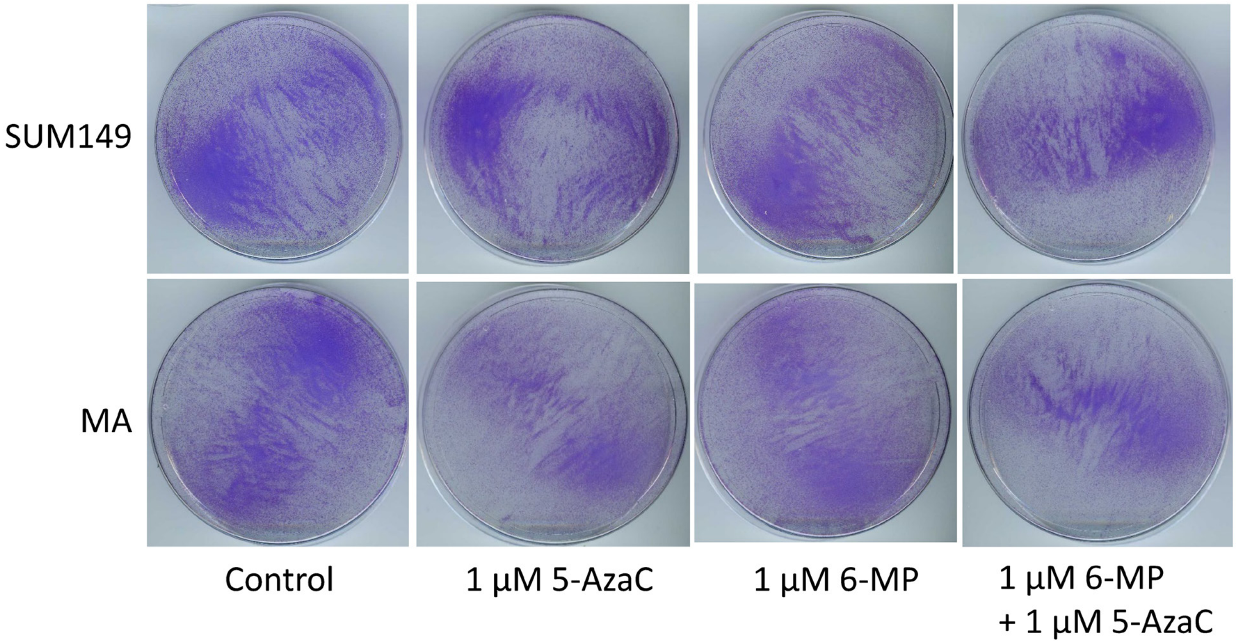

Supplementary Figure 1: A low dose of 6-MP does not significantly inhibit cell proliferation in 7 days. Parental SUM149-Luc (top panel) or metabolically adaptable (MA; bottom panel) cells were plated on 10-cm dishes and treated with 1 µM 5-AzaC, 1 µM 6-MP, or a combination of both drugs for 7 days. Cells in all dishes reached confluency to an extent similar to that of control untreated dishes. The dishes were stained with crystal violet.

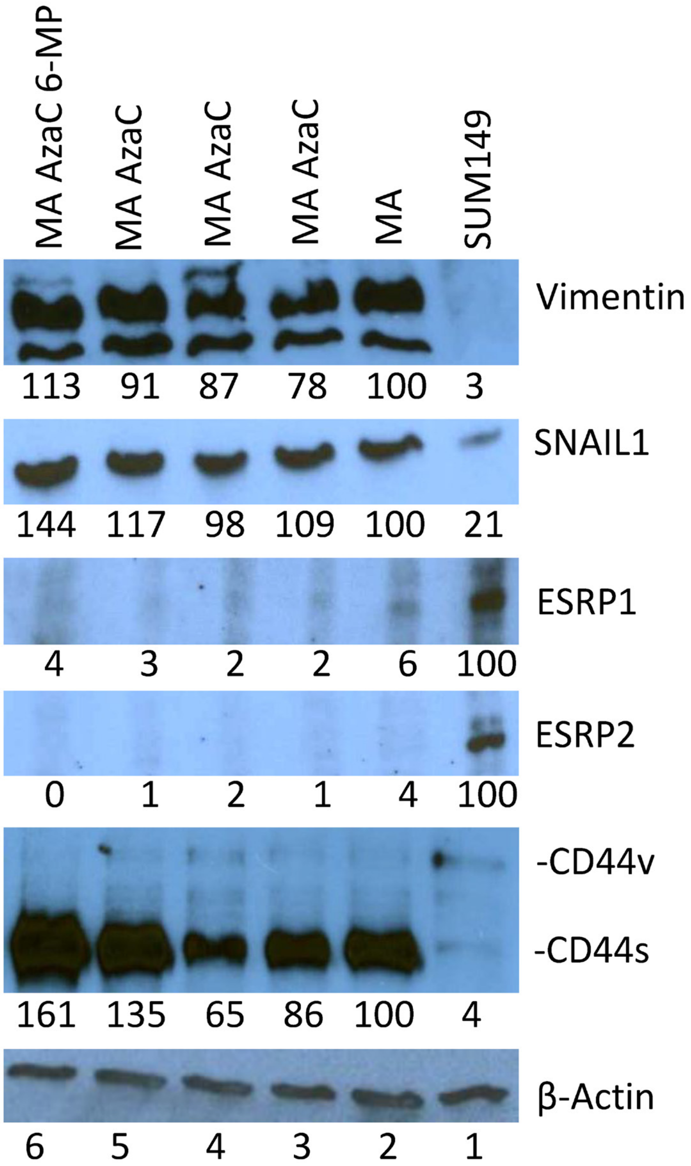

Supplementary Figure 2: Long treatments with 5-AzaC and 6-MP do not significantly affect markers of EMT in SUM149-MA cells. Parental SUM149-Luc cells were cultured in glutamine-containing medium with dialyzed fetal bovine serum (FBS; indicated in the figure as SUM149; lane 1). SUM149-MA cells (MA) were maintained in a glutamine-free medium with dialyzed FBS for nine passages and then switched to glutamine-containing medium for five passages before preparing cell lysates for this analysis (lane 2). MA cells were treated with drugs for long periods before preparing cell lysates for Western blotting. Lane 3: 1 µM 5-AzaC for 16 days, no drug for 8 days, and then 5-AzaC for 8 days. Lane 4: 1 µM 5-AzaC for 28 days and then no drug for 39 days. Lane 5: 1 µM 5-AzaC for 28 days and then no drug for 7 days. Lane 6: 1 µM 5-AzaC for 28 days, no drug for 7 days, 4 µM 6-MP for 24 days, and then no drug for 34 days. Western blotting was performed as described in Materials and Methods. Filters were re-probed with a β-actin antibody to normalize sample loading. The β-actin blot shown here is a re-probe of the CD44 blot. Control lanes 1 and 2 are identical to those shown in Figure 1. Relative intensities of protein bands, as quantified with the ImageJ software, are shown at the bottom; the values under the CD44 blot are for CD44s.
